# Rac1 regulates lipid droplets formation, nanomechanical, and nanostructural changes induced by TNF in vascular endothelium in the isolated murine aorta

**DOI:** 10.1007/s00018-022-04362-7

**Published:** 2022-05-27

**Authors:** Marta Z. Pacia, Natalia Chorazy, Magdalena Sternak, Benedikt Fels, Michal Pacia, Mariusz Kepczynski, Kristina Kusche-Vihrog, Stefan Chlopicki

**Affiliations:** 1 Jagiellonian Centre for Experimental Therapeutics (JCET), Jagiellonian University, 14 Bobrzynskiego Str., 30-348 Krakow, Poland; 2 Institute of Physiology, University of Luebeck, 160 Ratzeburger Allee, 23562 Luebeck, Germany; 3 Faculty of Chemistry, Jagiellonian University, 2 Gronostajowa Str., 30-387 Krakow, Poland; 4 Chair of Pharmacology, Jagiellonian University, 16 Grzegorzecka Str., 31-531 Krakow, Poland; 5 DZHK (German Research Centre for Cardiovascular Research), Partner Site Hamburg/Kiel/Lübeck, Lübeck, Germany

**Keywords:** Inflammation, Lipogenesis, ATGL, Raman imaging, Atomic force microscopy, Fluorescence microscopy, Scanning electron microscopy

## Abstract

Endothelial inflammation is recognized as a critical condition in the development of cardiovascular diseases. TNF-induced inflammation of endothelial cells is linked to the formation of lipid droplets, augmented cortical stiffness, and nanostructural endothelial plasma membrane remodelling, but the insight into the mechanism linking these responses is missing. In the present work, we determined the formation of lipid droplets (LDs), nanomechanical, and nanostructural responses in the model of TNF-activated vascular inflammation in the isolated murine aorta using Raman spectroscopy, fluorescence imaging, atomic force microscopy (AFM), and scanning electron microscopy (SEM). We analysed the possible role of Rac1, a major regulator of cytoskeletal organization, in TNF-induced vascular inflammation. We demonstrated that the formation of LDs, polymerization of F-actin, alterations in cortical stiffness, and nanostructural protuberances in endothelial plasma membrane were mediated by the Rac1. In particular, we revealed a significant role for Rac1 in the regulation of the formation of highly unsaturated LDs formed in response to TNF. Inhibition of Rac1 also downregulated the overexpression of ICAM-1 induced by TNF, supporting the role of Rac1 in vascular inflammation. Altogether, our results demonstrate that LDs formation, an integral component of vascular inflammation, is activated by Rac1 that also regulates nanomechanical and nanostructural alterations linked to vascular inflammation.

**Supplementary Information:**

The online version contains supplementary material available at 10.1007/s00018-022-04362-7.

## Background

Mechanistic insight into multiple changes at the biochemical, mechanical, and structural levels that occur inside the endothelium in vascular inflammation is important for understanding the mechanisms of cardiovascular diseases. Inflammation of endothelial cells (ECs) induced for example by a pro-inflammatory cytokine tumour necrosis factor (TNF) results in a broad spectrum of endothelial responses including the activation of the polymerization process of F-actin in ECs [1], decrease in elasticity parameter [1, 2], decrease in NO production [1], structural changes of ECs membrane with the formation of membrane protrusions [3, 4], activation of promotion of leukocyte trans-endothelial migration [5], upregulation of reactive oxygen species (ROS) [6], upregulation of intercellular and vascular cell adhesion molecules 1 expression (ICAM-1 and VCAM-1, respectively) [7], induction of cyclooxygenase-2 (COX-2) [7], Weibel–Palade bodies-dependent release of von Willebrand factor [7], de novo formation of lipid droplets (LDs), [8–10], and others. Nevertheless, the mechanism linking major TNF-induced aspects of endothelial inflammation is still elusive. In particular, it is not fully understood how the formation of LDs is linked to nanomechanical and nanostructural changes in endothelial inflammation and whether there is a central pathway that links these events.

Ras homologous (Rho) protein family, belonging to small guanosine–trisphosphate binding proteins (GTPases), are considered to be central regulators of actin reorganization and consequently function in cell migration, adhesion, polarity, membrane trafficking and cytokinesis [11, 12]. Interestingly, Rac1, one of the three best characterized members of the family, is postulated to act through several downstream targets to regulate cytoskeleton remodeling and F-actin accumulation at the leading edge of cells in the form of lamellipodia under physiological conditions [13]. Upon inflammation of ECs, Rac1 is required for TNF-induced cytoskeletal remodelling towards dense filament organization, and upregulation of F-actin polymerization [4], which is related to augmented endothelial stiffness [14, 15]. Moreover, the endothelial cytoskeleton has an established role in the release of vasoactive substances [16], due to the fact that endothelial NO-synthase (eNOS) is co-localized with cortical F-actin in the plasma membrane of endothelial cells, and polymerization of the F-actin actin filaments decreases eNOS activity/NO release, while the depolymerization has an opposite effect [17, 18]. Furthermore, the NADPH-dependent oxidases (NOX) activation and reactive oxygen species (ROS) generation is Rac1-dependent, as shown in ECs in vitro [19] and in Rac1-deficient mice [20]. The increased generation of ROS via activation of NOX upon TNF exposure has further implications for activation of the nuclear factor kappa-light-chain-enhancer of activated B cells [21], and results in overexpression of ICAM-1 [22]. Furthermore, inhibition of Rac1 function by a negative Rac1 mutant of airway epithelial cells [22] or endothelial cells [23] suppressed TNF-induced ROS generation [22] and ICAM-1 expression [22, 23], further supported  the role of Rac1 in inflammatory response.

LDs are defined as spherical cytoplasmic inclusions rich in triacylglycerols and cholesterols, surrounded by a phospholipid monolayer membrane including the proteins [24]. LDs were detected in a number of cell types. LDs biogenesis in the course of endothelial inflammation was recently suggested to represents an integral part of endothelial inflammation in LPS- [9] or TNF-stimulated ECs in vitro [8], or in the isolated vascular wall ex vivo [10]. However, the mechanisms of LDs formation in the endothelium upon vascular inflammation are unclear. Some authors suggested that LDs may represent an energy reservoir of the cell, a regulator of ECs glycolysis or a protector against excess lipids [25], yet, the pathway of LD biogenesis in endothelial inflammation have not been revealed.

In this work, we investigated whether Rac1 activation is involved in LDs biogenesis in endothelial inflammation as well as in mechanism linking LDs formation with nanomechanical and nanostructural alterations of the TNF-induced inflammatory state of ECs inside isolated blood vessels. Using the combination of techniques: Raman spectroscopy, fluorescence imaging, atomic force microscopy (AFM) and scanning electron microscopy (SEM) demonstrated that the formation of LDs, the polymerization of F-actin, changes in cortical stiffness, and nanostructural response to TNF stimulation were Rac1-dependent.

## Methods

Detailed and expanded methods are included in the Supplementary Information.

Briefly, the aorta were isolated from C57/BL/6 J mice, cleaned from the surrounding tissue, cut into rings and transferred to medium (minimal essential medium with the addition of 1% vitamins, 1% antibiotics (penicillin 10,000 U/ml and streptomycin 10,000 μg/mL), 1% non-essential amino acids and 20% fetal bovine serum). The aortic rings were then incubated in the absence or presence of murine TNF-α (10 ng/ml, 24 and 48 h; Sigma Aldrich, denoted in the manuscript as **TNF**), atglistatin (10 µM, 48 h; Cayman Chemical Company; denoted in the manuscript as **Atgl**), or Rac1 inhibitor: NSC23766 trichydrochloride (50 µM, 24 and 48 h; Sigma Aldrich; denoted in the manuscript as **NSC23766**).

For Raman, fluorescence, AFM, and SEM measurements en face aorta preparations were used as described before [10, 26]. Briefly, the resected and split-open aorta was tightly glued to the Cell-Tak^®^-coated surface. Subsequently, the tissue was preserved by a 10-min soak in formalin/paraformaldehyde for Raman, fluorescence (LDs, PECAM-1, and F-actin detection), AFM, and SEM imaging, while the non-fixed aorta was used for AFM-based nanoindentation measurements. Immunostaining of ICAM-1 expression was performed in cross sections of the aorta.

Raman and AFM imaging was carried out using a WITec Confocal Raman Imaging system (WITec alpha300, Ulm, Germany). Raman spectra of tissues were collected with the application of a 63 × water immersive objective (Zeiss Fluor, NA = 1.0), using the maximum laser power at the sample position (*ca.* 30 mW), a laser with the excitation wavelength of 532 nm, and 0.4 s exposure time per spectrum. The nominal minimal horizontal and vertical resolution for our setup is 0.32 and 0.53 μm, respectively, and sampling density of 0.40–0.50 and 1.0 μm in *x*/*y* and *z* direction, respectively, were used. The cortical stiffness of the endothelium was determined using an atomic force microscope (AFM; MultiMode SPM, Bruker, Germany) equipped with a feedback-controlled heating device (Nanoscope Heater Controller; Digital Instruments, Veeco, USA). To exclusively determine the stiffness of the endothelial cell cortex, soft triangular cantilevers (Novascan, USA) with a nominal spring constant of 0.03 N/m and a polystyrene sphere (10 µm) as a tip were used.

## Results

### Inhibition of Rac1 reduced the formation of LDs in TNF-activated endothelium in *en face* aorta

**Fig. 1:**
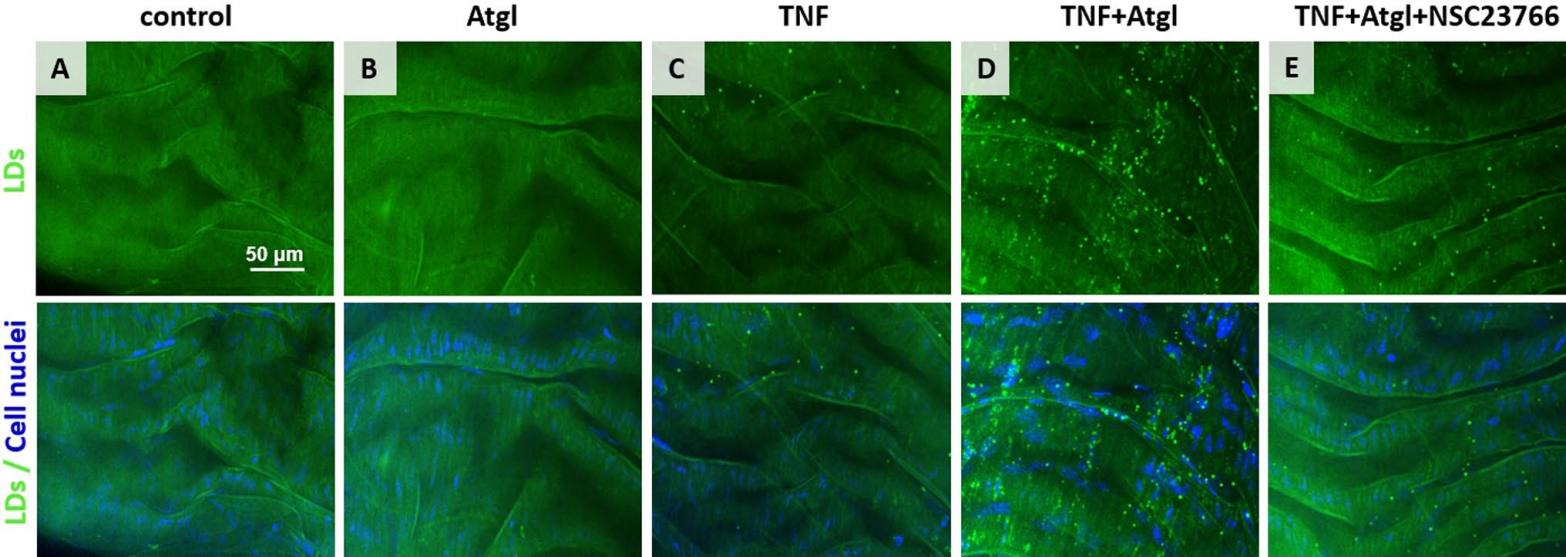
Formation of LDs in response to TNF in the absence or the presence of atglistatin, and/or NSC23766 in *en face* murine aorta preparation. Representative microphotographs of immunostaining of control *en face* aorta (**A**), aorta treated with Atgl (**B**; 10 µM, 48 h), TNF (**C**; 10 ng/ml, 48 h), TNF + Atgl (**D**; 10 ng/ml and 10 µM, respectively, 48 h), and aorta treated with TNF + Atgl + NSC23766 (**E**; 10 ng/ml, 10 µM and 50 µM, respectively, 48 h). Green (stain: BODIPY 493/503) and blue (stain: Hoechst 33,258) fluorescence originates from LDs and cell nuclei, respectively

Fluorescence imaging of LDs was performed in isolated blood vessels in response to TNF in the absence or the presence of atglistatin, and in the absence or the presence of NSC23766 (Fig. 1; LDs and nuclei visualized in the green and blue channel, respectively). In the case of murine thoracic aorta stimulated with TNF (10 ng/ml, 48 h), the formation of LDs in the endothelium was negligible (Fig. 1C). However, in the presence of atglistatin (inhibitor of adipose triglyceride lipase, ATGL), TNF resulted in the formation of numerous LDs in the endothelium (Fig. 1D) compared to the control. Atglistatin alone did not cause the formation of LDs (Fig. 1B). Therefore, in further studies to induce sustainable LDs formation in the aorta, TNF (10 ng/ml) and atglistatin (10 µM) were used together. To test whether the Rac1 was involved in the formation of LDs, we blocked Rac1 activity by NSC23766, which resulted in a significant reduction in LDs formation in aorta samples stimulated with TNF (in the presence of atglistatin and NSC23766, TNF + Atgl + NSC23766; Fig. 1E).

**Fig. 2:**
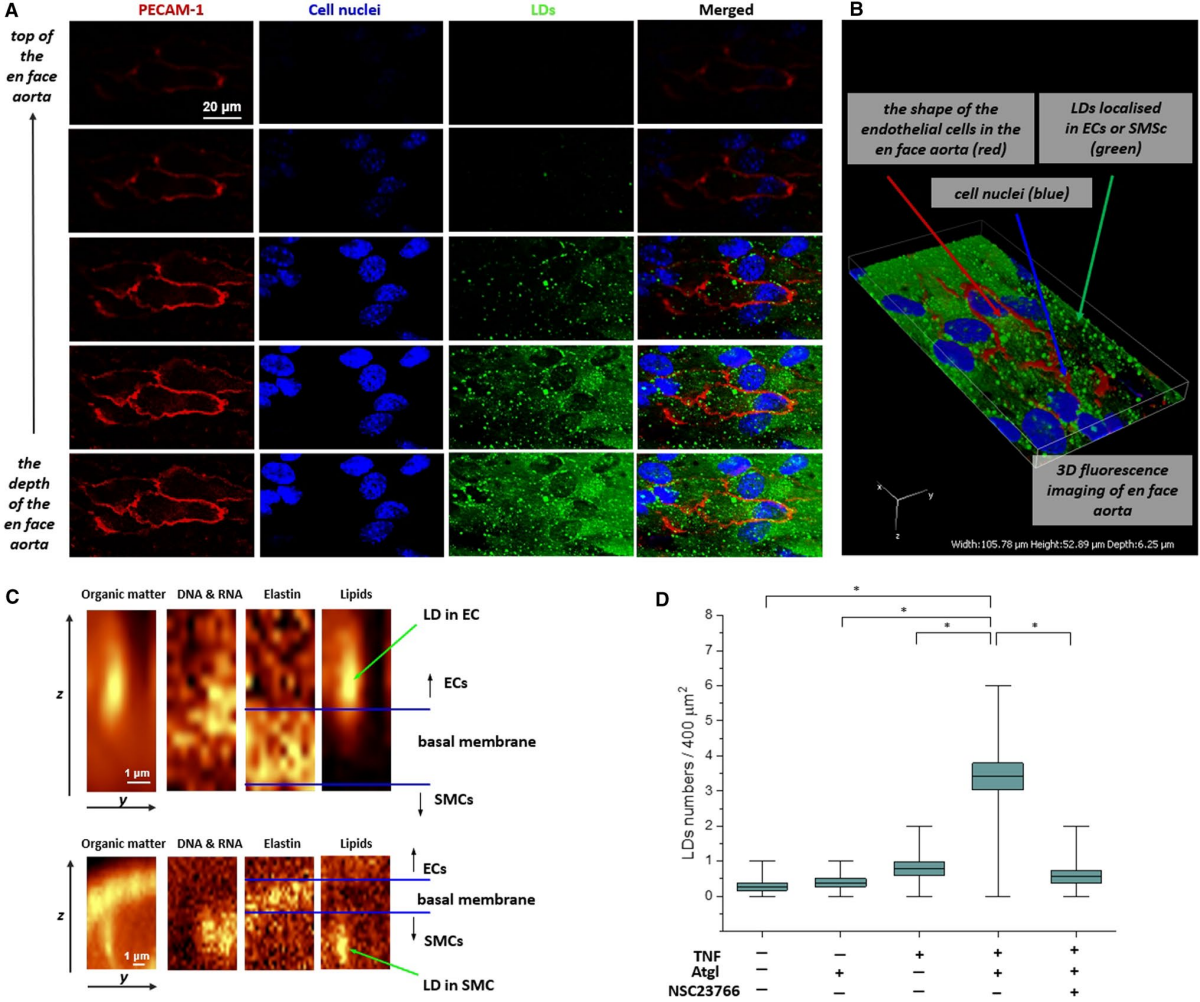
Localization of LDs in the aortic vascular wall in *en face* aorta stimulated by TNF in the presence of atglistatin. Confocal fluorescence microscopy z-stack images of fragment of *en face* aorta stimulated by TNF + Atgl (10 ng/ml and 10 µM, respectively, 48 h). Z stack images were acquired from the top to the depth of the *en face* aorta with 0.33-μm layer separation (**A**), and reconstructed as a 3D image (**B**). Red, blue and green fluorescence originate from PECAM-1 (stain: Cy3-antibody), cell nuclei (stain: Hoechst 33,258) and LDs (stain: BODIPY 493/503), respectively. 3D perspective Raman images of *en face* aorta showing the distribution of organic matter (integration in the 2800–3100 cm^−1^ range), DNA and RNA (778–808 cm^−1^), elastin (500–550 cm^−1^) and lipids (2830–2900 cm^−1^) in aorta stimulated by TNF + Atgl (**C**; 10 ng/ml and 10 µM, respectively, 48 h). Due to the presence of elastin fibres indicating the localization of the basal membrane, the population of LDs was separated into those LDs localised in the endothelium (**C**, upper panel), and LDs belonging to SMCs (**C**, lower panel). The calculations of number of endothelial LDs per one Raman image was performed (the typical size of Raman image 20 × 20 µm; **D**)

The presented images of the LDs fluorescence in the aorta *en face* give a general picture of the presence and number of lipid droplets under various control conditions (Fig. 1). As the presented images were recorded using a lens with a 40 × magnification and a numerical aperture of 0.6, the vertical resolution is about 2 µm, which makes it difficult to unambiguously assign LDs to their location: endothelial cells or smooth muscle cells. To estimate the number of LDs divided into LDs in the endothelium and in the smooth muscle, 3D Raman imaging was used (Fig. 2).

Also, the PECAM-1 images were recorded for aorta samples in the presence of atglistatin, TNF, TNF in the presence of atglistatin (TNF + Atgl), or TNF in the presence of atglistatin and NSC23766 (TNF + Atgl + NSC23766). In immunohistochemical staining, PECAM-1 is used primarily to demonstrate the presence of endothelial cells and visualisation of the shape of EC based on endothelial cell intercellular junctions. The images of PECAM-1 staining did not show any visible differences in comparison to control, indicating the preserved integrity of the endothelial layer in the blood vessel in all studied conditions (Fig. S1).

### Confirmation of localization of LDs in endothelium in isolated murine aorta

To confirm that LDs were located in the endothelium, 3D fluorescence and Raman imaging were provided (Fig. 2). Fluorescence z-stack images acquired from the top to the bottom with 0.33-μm layer separation of *en face* aorta stimulated by TNF + Atgl (10 ng/ml and 10 µM, respectively, 48 h) confirmed the presence of LDs in the endothelial layer of the aortic wall. This was evidenced by the mutual position of the signals from the LDs and the PECAM-1, indicating endothelial cell intercellular junctions [10, 25] (Fig. 2A, B).

Fluorescence imaging as well as confocal Raman spectroscopy/imaging are two well-established methods providing insight into the compartments of cells and tissues. While fluorescence imaging allows for an overall picture of the tissue and, for example, to assess the abundance of LDs, it requires the use of dye molecules or a fluorescent protein. It is also difficult to distinguish the molecular structure of lipids by fluorescence. Raman imaging solves the problem of label-free, non-destructive intracellular measurements of biochemical composition of lipids [26–30]. Due to the large Raman scattering cross section for lipids, bands originating from lipids can be clearly detected in Raman spectra. It enables not only the detection of lipid structures inside endothelium/tissues (e.g., lipids droplets in isolated en face aorta activated by TNF or oleic acid) but also determination of LDs chemical composition [10, 26]. The short description with visual explanation of Raman spectroscopy/Raman imaging principles is presented in the Supplementary Materials (Fig. S2). Due to the combination of a Raman spectrometer with a confocal microscope, and the use of an objective with a magnification of 60 × and a numerical aperture of 1, it is possible to image tissues with a vertical resolution of about 0.5 µm; however, as sampling density in z-axis was 1.0 µm, the z-resolution is roughly of this order.

Figure 2C presents Raman images of all organic compounds obtained by integrating the intensity of the C−H stretching vibrations in the 2800−3100 cm^−1^ spectral range, nucleic acids in the 778−808 cm^−1^ range, and elastin fibres in the 500–550 cm^−1^ range [31]. The Raman image, obtained by the integration of bands characteristic for all lipids in the 2830−2900 cm^−1^ range [32, 33], shows the distribution of newly formed LDs inside fragments of aortic tissues stimulated by TNF in the presence of atglistatin (10 ng/ml and 10 µM, respectively, 48 h). The longitudinal shape of LDs presented in the z-stack Raman measurements was the consequence of vertical resolution of confocal Raman microscopy, and did not reflect the real round shape of LDs (Fig. 2C). Due to the presence of elastin fibres indicating the localization of the basal membrane, the population of LDs localised in the endothelium (Fig. 2C, upper panel), and LDs belonging to SMCs (Fig. 2C, lower panel) were identified. As it was undoubtedly visible in Fig. 2C, LDs were placed above or below the basal membrane, which was considered as a clear-cut border between the ECs and SMCs in the aortic vessel wall. This discrimination of LDs in ECs and SMCs allowed for a separate Raman analysis of the number of LDs (Fig. 2D) or biochemical composition of LDs depending on their localisation.

### Distinct chemical characterisation of LDs formed in endothelial and smooth muscle cells in *en face* aorta stimulated by TNF in the presence of atglistatin

**Fig. 3:**
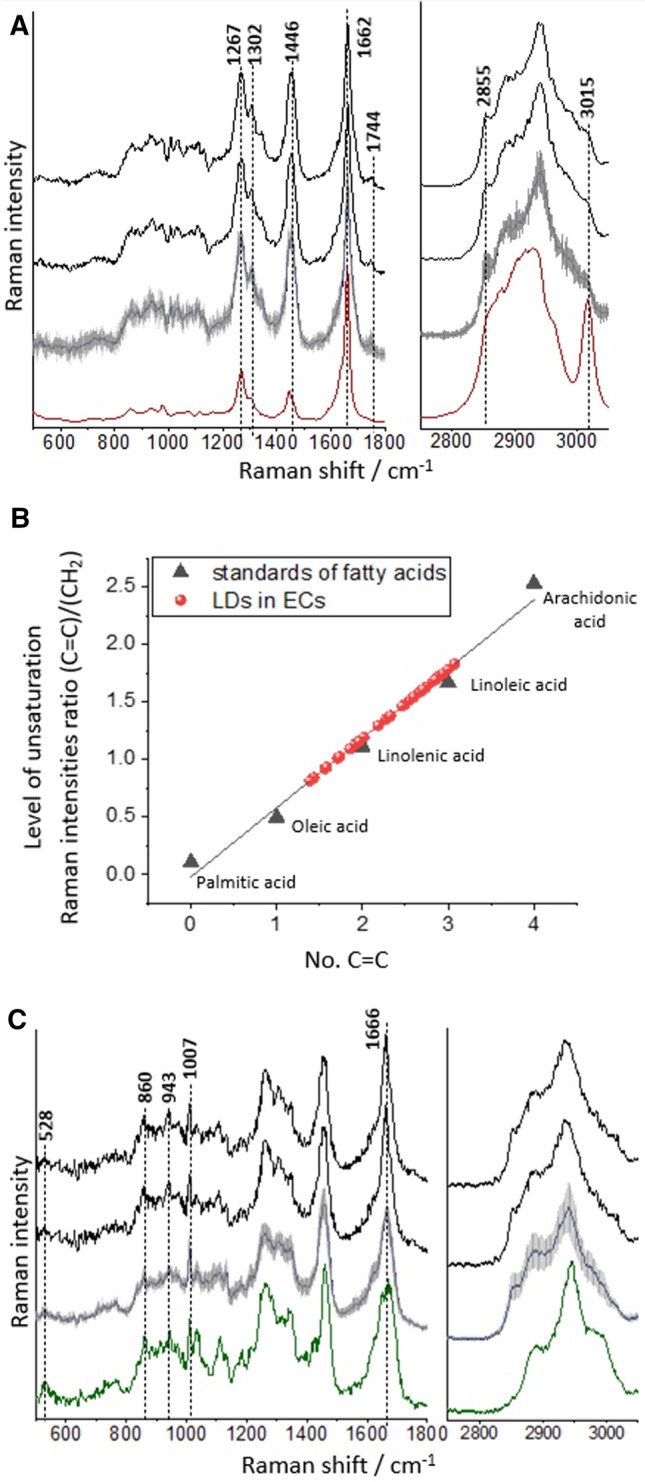
Chemical characterisation of LDs formed in the endothelium or in smooth muscle cells in *en face* aorta stimulated by TNF in the presence of atglistatin (10 ng/ml and 10 µM, respectively, 48 h). Representative Raman spectra of LDs extracted from ECs measured within the blood vessel (black spectra) and averaged over all measured cells (blue spectrum; spectrum presented with the standard error on each data point), with comparison to Raman spectrum of arachidonic acid (**A**; red spectrum). The calibration curve of fatty acids showing heterogeneity of individual level of unsaturation of endothelial LDs (**B**; representative Raman spectra of LDs extracted from SMCs measured within the blood vessel (black spectra) and averaged over all measured cells (blue spectrum; spectrum presented with the standard error on each data point), with comparison to Raman spectrum of elastin (**C**; green spectrum)

Raman spectroscopy allows the measurement of small areas of tissue with high accuracy, which allows the registration of Raman spectra of LDs, but this approach requires samples with a large number of LDs (Fig. 3). During measurements, the Raman signal was recorded between 0.40 and 0.50 µm (objective 60 × magnification and NA = 1). Analysis of biochemical characterization of LDs using Raman spectroscopy was performed only for the aorta stimulated by TNF in the presence of Atgl, where numerous LDs were observed.Therefore, for the remaining groups, including the aorta stimulated only with TNF, or only with Atgl, where single LDs were observed, Raman analysis of LDs was not possible and was not performed.

To study the composition of LDs formed in ECs or SMCs in response to TNF, the single Raman spectra were extracted from the core of each LDs and then averaged. Such obtained single spectra (with the best quality), and average Raman spectra (with denoted standard error of mean showing heterogeneity) of LDs localised in ECs or SMCs are presented in Fig. 3A or C, respectively. Based on a comparison between spectra of endothelial LDs (Fig. 3A) and arachidonic acid (Fig. 3A, red spectrum), the Raman spectra of endothelial LDs were assigned mostly to the lipid unsaturated components. We decided to assess the similarity of the Raman signature of LDs spectra with the spectrum of arachidonic acid (AA), as AA is considered to be the substrate for the de novo formation of endothelial LDs. The assignment of the band at *ca*. 1744 cm^−1^, attributed to the carbonyl stretching vibrations [32] indicated that the main component of LDs were triacylglycerols, not free fatty acids. LDs contained negligible content of cholesterols and phospholipids based on the barely noticeable intensity of bands at *ca*. 702 and 721 cm^−1^ originating from the cholesterol ring deformations and the symmetric stretching vibrations of the N^+^(CH_3_)_3_ choline group, respectively [33].

The standard error for averaged spectra of endothelial LDs was rather small (grey shadow in blue spectrum in Fig. 3A) what indicated the general homogeneity in the chemical composition of LDs; however, the intensities of the bands connected with level of unsaturation varied. In the Raman spectra of lipids, the level of unsaturation manifested itself by the presence the higher intensity of bands at 1267, 1662 and 3015 cm^−1^, assigned to the in-plane C=C–H bending, C=C stretching and =C–H stretching modes, respectively [32]. The degree of unsaturation can be determined as the n(C = C)/n(CH_2_) intensity ratio obtained by integration of the respective Raman bands (1662/1446 cm^–1^) and fitted to a calibration curve for unsaturated fatty acids (Fig. 3B) [26, 27]. The results showed that endothelial LDs formed upon stimulation with TNF (in the presence of Atgl) were rich in highly unsaturated lipids with an estimated number of C=C bonds for lipids inside LDs in the range of 1.39–3.07 (average value equalled 2.4 ± 0.1). Moreover, a slight trend pointing to the relationship between the endothelial LDs individual level of unsaturation with their sizes was found: the smaller endothelial LDs were, the higher was the level of unsaturation of lipids building LDs (Fig. S3).

The representative Raman spectra of LDs localised in SMCs, in comparison to the Raman spectrum of elastin (green spectrum) are presented in Fig. 3C. Although the Raman signal of LDs in SMCs had a unique Raman profile that allowed them to be recognized among the other designated structures present in the aortic tissue, theirs Raman spectra contained features also derived from proteins. Occurrence of bands at *ca*. 1007 (phenylalanine) [34], 943 (N–C–C in the structure of proteins) [34], 860 (tyrosine) [34], and 528 cm^−1^ (S–S stretching vibrations of disulphide bonds in the elastin structure) [35], as well as the general broader shape of bands at *ca*. 1666 and 2940 cm^−1^ indicated the presence of protein components. It is presumed that the Raman signal of proteins in the spectra of LDs in SMCs came mainly from elastin fibres, as the basal membrane, composed mostly of elastin, was a border between ECs and SMCs.

### Inhibition of Rac1 decreased the cortical stiffness in TNF-activated endothelium in *en face* aorta

**Fig. 4:**
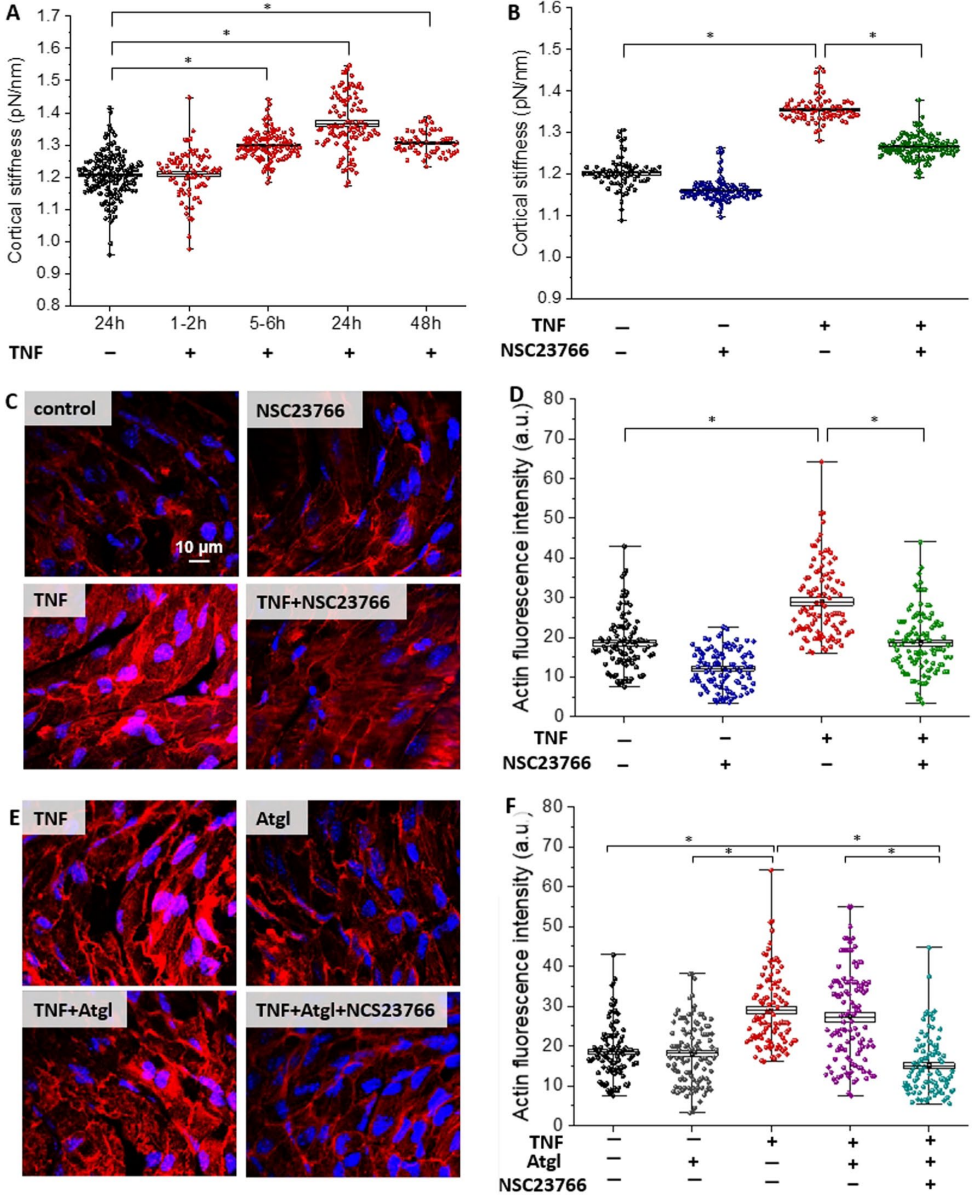
Changes in cortical stiffness and F-actin architecture in ECs in aorta *en face* stimulated by TNF. The time-dependent changes in the cortical stiffness of ECs stimulated by TNF (10 ng/ml) determined using AFM: control mice (*N* = 8, *n* = 160), and mice treated with TNF for 1–2 (*N* = 6, *n* = 118), 5–6 (*N* = 6, *n* = 118), 24 (*N* = 6, *n* = 101), or 48 h (*n* = 4, *n* = 52), respectively (**A**). Changes in cortical stiffness (**B**) of ECs within aorta stimulated by TNF (10 ng/ml, 24 h; *N* = 5, n = 70), and TNF in the presence of NSC23766 (TNF + NSC23766, 10 ng/ml and 50 µM, respectively, 24 h; *N* = 5, *n* = 135) in comparison to the control (*N* = 5, *n* = 72), and NSC23766 (50 µM; *N* = 5, *n* = 130). Representative microphotographs (**C**, **E**) of staining of control *en face* aorta (*N* = 3, *n* = 101), and aorta treated with NSC23766 (50 µM, 24 h; *N* = 3, *n* = 101), TNF (10 ng/ml, 24 h; *N* = 3, *n* = 98), TNF + NSC23766 (10 ng/ml and 50 µM, respectively, 24 h; *N* = 3, *n* = 102), Atgl (10 µM, 24 h; *N* = 3, *n* = 105), TNF + Atgl (10 ng/ml and 10 µM, respectively, 24 h; *N* = 3, *n* = 110), TNF + Atgl + NSC23766 (10 ng/ml, 10 µM and 50 µM, respectively, 24 h; *N* = 3, *n* = 92); red and blue fluorescence originating from F-actin (stain: phalloidin-TRITC) and cell nuclei (Hoechst 33,258), respectively. Quantification of F-actin fluorescence intensity of studied groups of samples (**D**, **F**). Values given as mean ± SD are shown in box plots: mean (horizontal line), SD (box), minimal and maximal values (whiskers)

To characterize the nanomechanical properties of ECs within *en face* aorta stimulated by TNF, the AFM was used. Force–distance curves analysis allowed for studying the effects of structural changes of the sub-membranous F-actin architecture on TNF response, and characterizing the cortical stiffness of ECs. In general, a two-stage response in endothelial cortical stiffness was evoked by TNF stimulation: a short incubation with the cytokine (1–2 h) did not cause changes (1.22 ± 0.07 pN/nm), while longer incubation (5–6, 24 or 48 h) resulted in significantly increased cortical stiffness of aortic ECs (1.30 ± 0.04, 1.37 ± 0.09, and 1.31 ± 0.04 pN/nm, respectively) in comparison to unstimulated cells (1.21 ± 0.07 pN/nm). However, stiffness after 48 h was reduced in relation to 24 h of incubation (Fig. 4A), thus 24 h of incubation with TNF was considered representative for augmented endothelial cortical stiffness linked to vascular inflammation (Fig. 4B).

The elastic properties of the cells are mainly determined by the cytoskeleton architecture and its microfilament: F-actin (filamentous actin), hence, ECs within en face aorta were stained for F-actin fibres and nucleic acids (red and blue channel, respectively). The images of phalloidin-stained TNF-stimulated ECs confirmed the formation of thicker bundles and induction of F-actin polymerisation, as evidenced by higher phalloidin-specific fluorescence intensity (Fig. 4C). This result confirmed that TNF-induced cortical stiffness was linked to cytoskeleton reorganization. Accordingly, an increased actin polymerization in the TNF-activated ECs was quantified and compared to the control using phalloidin stainings for F-actin (28.98 ± 0.94 arbitrary units, *vs*. 18.49 ± 0.69 a.u., respectively; Fig. 4D).

To test whether the Rac1 was involved in alterations in nanomechanical properties of ECs inside isolated blood vessels in response to TNF, we blocked Rac1 activity by NSC23766, which resulted in a significant decrease in cortical stiffness in ECs stimulated with TNF + NSC23766 (1.26 ± 0.03 pN/nm), in comparison to TNF alone (1.35 ± 0.03 pN/nm; Fig. 4B), as well as resulted in a significant decrease in actin fluorescence intensity in ECs stimulated with TNF + NSC23766 (18.50 ± 0.75 a.u.), in comparison to TNF alone (28.98 ± 0.94 a.u.).

Based on the close correlation between the stiffness of endothelial cells (Fig. 4B) and the polymerized F-actin (based on quantification phalloidin fluorescence intensity; Fig. 4D), we used this correlation to evaluate changes in the endothelial cytoskeleton in the aorta samples treated with atglistatin, TNF + Atgl, or TNF + Atgl + NCS23766 (Fig. 4E, F). Quantification of the fluorescence intensity of phalloidin-stained ECs confirmed that atglistatin did not cause the changes in the F-actin architecture itself, as there was no change between the control aorta and aorta treated with atglistatin. Similarly, there was no difference between aorta stimulated with TNF alone and TNF together with atglistatin. Moreover, atglistatin did not alter the decreasing F-actin fluorescence intensity for aorta stimulated by TNF + Atgl + NSC23766 in comparison to aorta stimulated by TNF + Atgl (Fig. 4F).

### Inhibition of Rac1 affected nanostructural alterations in TNF-activated endothelium in en face aorta

**Fig. 5:**
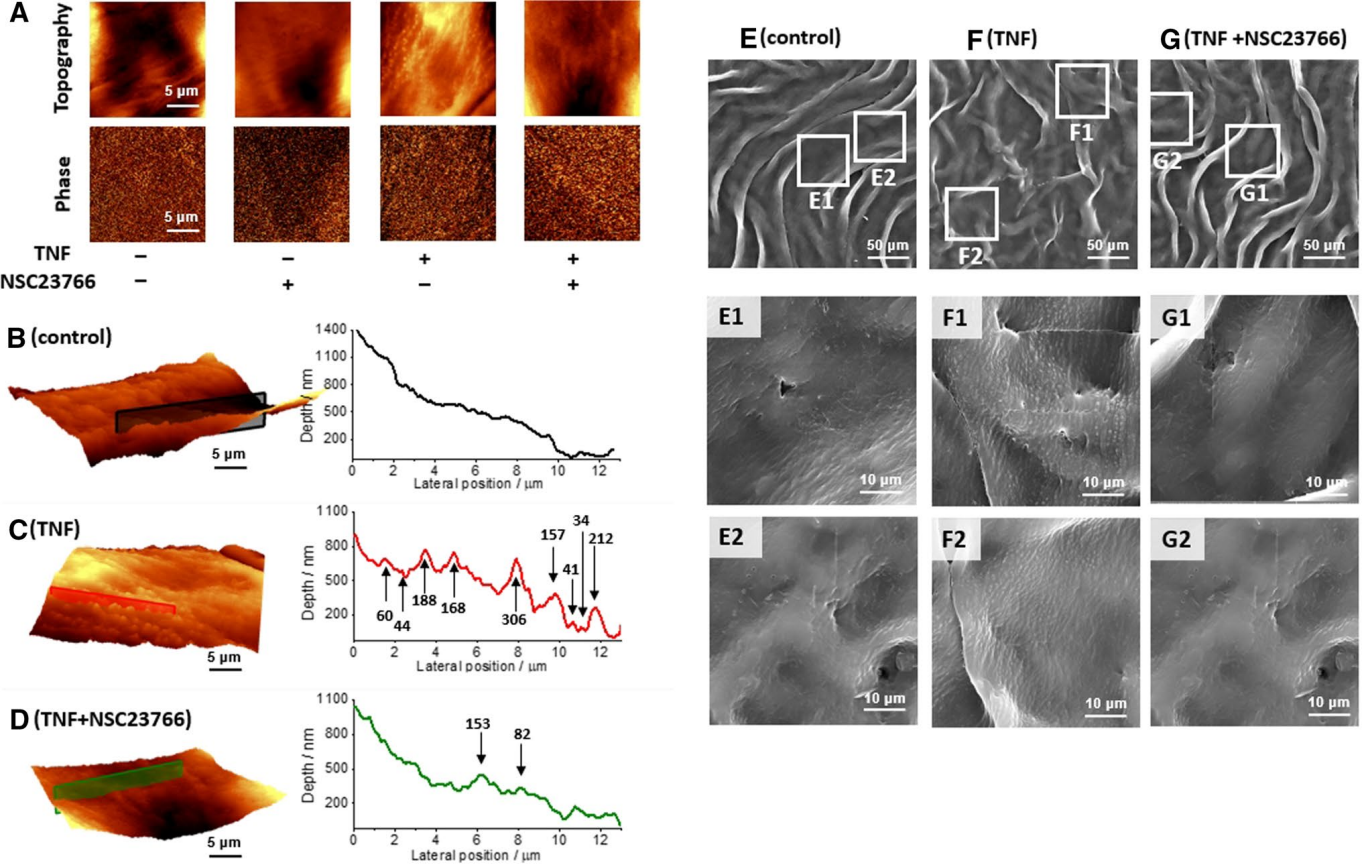
Nanoscale alterations of the surface of activated ECs in *en face* aorta stimulated by TNF, in the absence and presence of NSC23766. Representative topography and phase AFM images of control en face aorta (**A**), aorta treated with NSC23766 (50 µM, 24 h), TNF (10 ng/ml, 24 h), and TNF + NSC23766 (10 ng/ml and 50 µM, respectively, 24 h). The 3D perspective of AFM images and detailed topography cross section of control *en face* aorta (**B**), aorta treated with TNF (**C**; 10 ng/ml, 24 h), and TNF + NSC23766 (**D**; 10 ng/ml and 50 µM, respectively, 24 h). Representative SEM images (size of images: 250 × 250 µm) of control aorta (**E**), TNF-activated aorta (**F**, 10 ng/ml, 24 h), and TNF + NSC23766 (**G**; 10 ng/ml and 50 µM, respectively, 24 h) with magnifications (size of images: 50 × 50 µm) showing detailed nanostructural properties of a control (E1–E2), TNF-activated ECs (F1–F2), and TNF + NSC23766 (G1–G2)

The endothelial barrier has a central role in inflammation, so the nanomechanical and nanostructural properties undergo modifications upon stimulation with TNF (10 ng/ml, 24 h). On the surface of TNF-activated ECs inside the blood vessels, we repeatedly observed nanomechanical alterations by AFM imaging, in comparison to the control; however, they were absent in ECs stimulated with TNF in the presence of the Rac1 inhibitor (NSC23766; Fig. 5A–D). The topography images indicated the existence of the protuberances on the surface of ECs, while the phase images confirmed that these structures had properties similar to the rest of the tissue. In the topography images, these assemblies were observed as protuberances of the endothelium surface with a height of approx. 20–400 nm (Fig. 5C).

The AFM imaging provided detailed an analysis of nanostructural properties after stimulation with TNF. However, due to some technical limitations, AFM measurements allowed for measurements of fragments of the en face aorta (in this case: 15 × 15 µm per image), which represented a small area of interest, in comparison to scanning electron microscopy imaging (SEM). SEM imaging confirmed the existence of nanostructural protuberances in the large region (250 × 250 µm per image) of TNF-activated ECs inside isolated blood vessels, in comparison to the control (Fig. 5E, F).

### Inhibition of Rac1 downregulated the overexpression of ICAM-1 in TNF-activated endothelium in cross section of aorta

**Fig. 6:**
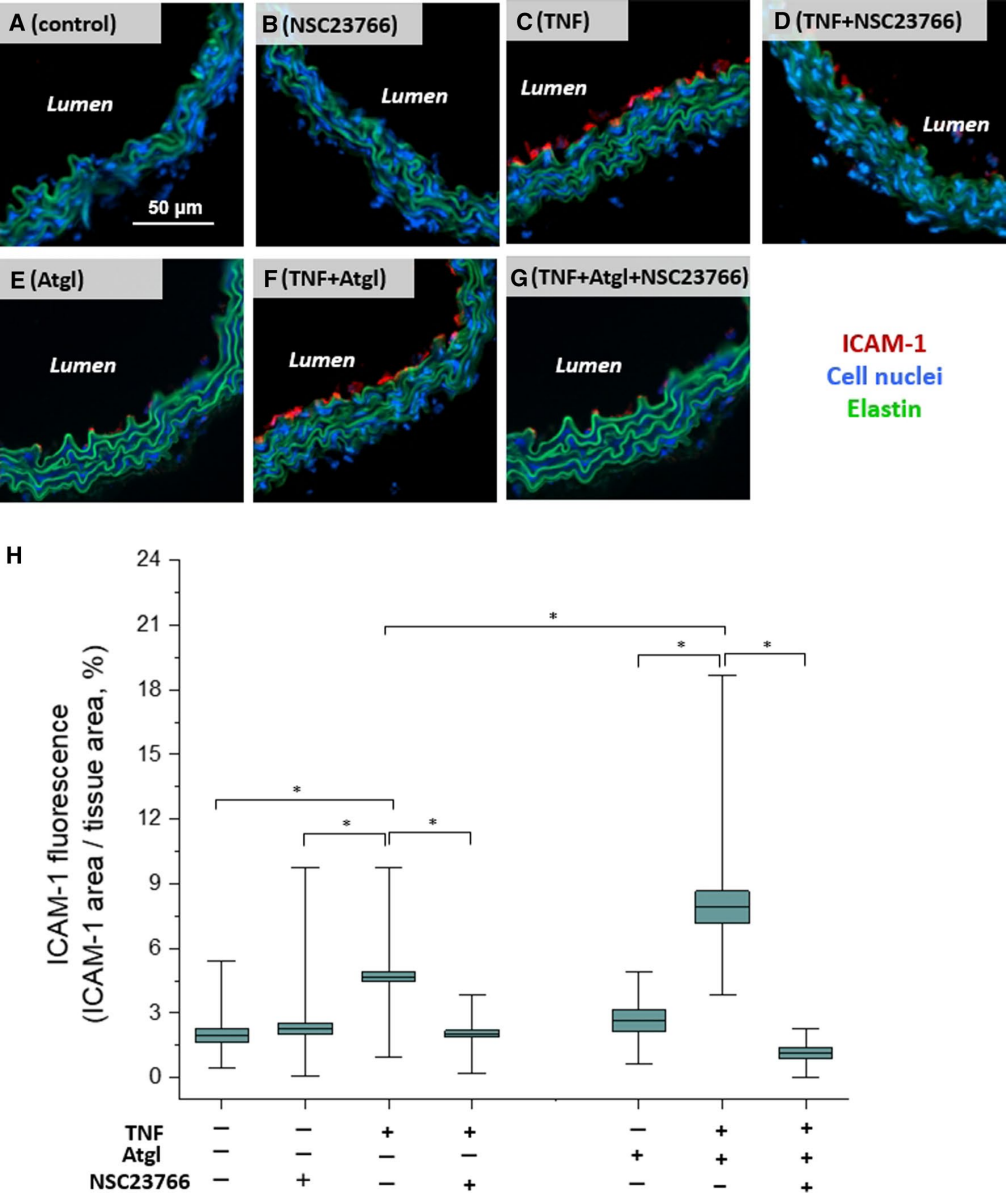
Expression of ICAM-1 in the cross section of aorta activated by TNF, in the absence and presence of NSC23766, or atglistatin. Representative microphotographs of ICAM-1 expression in cross section of control aorta (**A**), aorta treated with NSC23766 (**B**; 50 µM, 24 h), TNF (**C**; 10 ng/ml, 24 h), TNF + NSC23766 (**D**; 10 ng/ml and 50 µM, respectively, 24 h), atglistatin (**E**; 10 µM, 24 h), TNF + Atgl (**F**; 10 ng/ml and 10 µM, respectively, 24 h), and TNF + Atgl + NSC23766 (**G**; 10 ng/ml, 10 µM and 50 µM, respectively, 24 h). Red, blue, and green fluorescence originate from ICAM-1 (stain: Cy3-antibody, cell nuclei (stain: Hoechst 33258), and elastin fibres (autofluorescence), respectively. Quantification of ICAM-1 expression of studied groups of samples, presented as the ratio of positively-stained ICAM-1 area to the total area of the tissue (**H**). Values given as mean ± SD are shown in box plots: mean (horizontal line), SD (box), minimal and maximal values (whiskers)

To confirm that endothelial alterations described above including the formation of LDs, nanomechanical and nanostructural alterations (stiffness, nanostructural protuberances), were all associated with the ongoing inflammation of ECs within *en face* aorta, the expression of ICAM-1, the typical marker for endothelial inflammation was assessed and quantified (Fig. 6). A representative fluorescence images of ICAM-1 (red), nuclei (blue), and elastic fibres (green) showed the increased expression of ICAM-1 in TNF-activated ECs (4.67 ± 0.21%; Fig. 6C), and in ECs stimulated with TNF in the presence of atglistatin (7.91 ± 0.74%; Fig. 6F), in comparison to the control samples (1.95 ± 0.29%; Fig. 6A). Neither the NSC23766 itself (2.26 ± 0.26%; Fig. 6B) nor atglistatin alone (2.65 ± 0.50%; Fig. 6E) increased ICAM-1 expression. However, in the presence of NSC23766, an increased expression of ICAM-1 in TNF-activated ECs (2.04 ± 0.16%; Fig. 6D), as well as in TNF + Atgl-activated ECs was blunted (1.14 ± 0.26%; Fig. 6G).

## Discussion

In the present work, we demonstrated that in the isolated murine vessel stimulated with TNF, a reliable model of vascular inflammation, biogenesis of LDs was mediated by Rac1-dependent pathway and was correlated to parallel changes in nanomechanical and nanostructural properties of endothelial cells including augmented cortical stiffness, cytoskeleton remodelling, and protuberances in the nanostructure of endothelial surface. In the present work, we not only underscored the indispensable role of Rac1 in the dynamic formation of LDs in endothelial inflammation but also a tight control of LDs degradation by ATGL. Indeed, the chemical composition of LDs in TNF-induced endothelial inflammation in the aorta could be studied in detail only in the presence of ATGL inhibition to prevent dynamic disappearance of LDs in the stimulated endothelium by a pro-inflammatory cytokine.

The major finding of this work was the demonstration that Rac1 signalling is involved in the formation of LDs (Fig. 1D). Of note, the effect of Rac1 on arachidonic acid (AA) turnover via phospholipase A_2_ (PLA_2_) activation in response to a variety of stimuli, including cytokines was reported in the literature [36–39]. Thus, we have hypothesized that the high level of unsaturation of LDs in TNF-activated endothelium (Fig. 3A, B), could be linked to the involvement of Rac1–PLA_2_–AA cascade in the formation of LDs and eicosanoid biosynthesis in the vascular wall. In previous studies, LDs formation was linked to PGI_2_ generation [9, 10, 25] but the details linking LDs formation and eicosanoid production in LDs in the vascular wall has not delineated as yet, in contrast to the well-known role of LDs in leukocyte in the generation of eicosanoids [40, 41]. Our results suggested the instrumental role of Rac1 in endothelial LDs formation that could be also linked to increased generation of eicosanoids in endothelium known to be regulated by the RhoA–Rac1–Cdc42 pathway [42].

It was important to show that degradation of LDs within aortic tissue was a highly dynamic process as blocking of LDs degradation by ATGL inhibitor was mandatory to study the LDs biochemical contents in more detail, otherwise, LDs were virtually absent in TNF-activated endothelium. These results might explain the reason why pro-inflammatory stimuli such as interleukin-1β or lipopolysaccharides did not cause significant LDs formation in the vascular wall in our previous study [10]. In the contrary to measurement of ECs in vessel setup, when the HMEC-1 cells (in vitro measurements) were exposed to TNF, the numerous and stable LDs were observed and the blockade of lipolysis using Atgl was not necessary to observe multiple LDs using fluorescence (Fig. S4). Therefore, the relationship between the formation and lipolysis of endothelial LDs in cell culture was different than in the isolated vessels underscoring the value of our approach to study LDs formation in endothelium in situ in the isolated aorta.

Here taking advantage of ATGL inhibitor, we inhibited LDs degradation in endothelium in isolated blood vessels and used Raman imaging to demonstrate that the reservoir of endothelial LDs included mainly LDs rich in highly unsaturated lipids with the estimated number of C=C bonds for lipids inside LDs in the range of 1.39–3.07 (average value equalled 2.4 ± 0.1), and negligible content of cholesterols and phospholipids (Fig. 3A, B). Our previous results indicated that the heterogeneity in the chemical composition and the level of lipid unsaturation of LDs in activated endothelium could be considered as a parameter differentiating the mechanism of LDs formation [8–10, 26, 27]. In particular, comparing the highly unsaturated chemical composition of endothelial LDs formed as a result of inflammation in the present study (evidenced here by ICAM-1 overexpression; Fig. 6) with LDs features linked to apoptosis induced by Fas ligand [10] (featured by unsaturated lipids with an average number of C=C bonds equalled 1.7, or rich in cholesterols with an average number of C=C bonds equalled 0.95) [26], underscored that the high level of endothelial LDs unsaturation could be recognized as a hallmark of vascular inflammation. The above-mentioned studies were conducted on ECs in situ within isolated blood vessels, what provided the natural environment and 3D intercellular interactions between the endothelial cells and other types of cells in the intact isolated vessel. Nevertheless, the results were in agreement with the previous research conducted on endothelial cell culture lines when also newly formed LDs in endothelium stimulated by TNF or LPS were characterized by a high level of lipid unsaturation [8, 9].

An interesting finding of this work to detect that TNF-induced LDs (in the presence of atglistatin) were formed not only in the endothelium but also in the smooth muscle cells (Fig. 2), indicating either the independent formation of LDs in the SMCs or their transfer from the ECs to the SMCs [10, 25]. Although the Raman signal of LDs in SMCs had a unique Raman profile that allowed them to be recognized among the other designated structures present in the aortic tissue, their Raman spectra contained features also derived from proteins. From a methodological point of view, to collect the Raman signal from LDs located in SMCs, the laser light must have passed through the elastin fiber, what resulted in scattered light originating from both LDs and elastin. As a consequence of a disturbance of the LDs Raman signature by a signal from elastin fibers we were not able to analyze the degree of unsaturation of the LDs localized in SMCs.

Increased stiffness of ECs cortex is considered as a hallmark of endothelial dysfunction [43, 44] and has been reported to occur in response to the mineralocorticoid hormone aldosterone [45], increased Na^+^ intake [46] or inflammation induced by TNF [1]. In general, the mechanical flexibility of the endothelial cortex relies on the reorganization of the structure of the cytoskeleton [47]. Shifting from depolymerized G-actin to polymerized F-actin (red fluorescence in Fig. 4C, E) caused increased stiffness of cortex of TNF-activated endothelium (Fig. 4A, B), and as shown here depended on the activity of the Rac1 (Fig. 4B). Although the relationship between cortical stiffening, via reorganization of the cytoskeleton, with members of the Ras superfamily of small GTPases including Rac1 was previously known, we correlated this response to LD formation suggesting the central role of Rac1 in the orchestrating LDs formation with nanomechanical and nanostructural alterations of the TNF-induced inflammatory state of ECs inside isolated blood vessels.

In fact, both the formation of LDs (via AA release via PLA_2_ from cell membrane [37]) and increased cortical stiffness in the TNF-activated ECs were accompanied by alterations on the cell surface, which have been studied using advanced imaging techniques: atomic force (Fig. 5A–D) and scanning electron microscopy (Fig. 5E–G). In the AFM topography images, the protuberances of the endothelium surface with a height of approx. 20–400 nm (Fig. 5C), were observed, while they were absent for ECs stimulated with TNF in the presence of NSC23766. The presence of nanoscale protuberances reported during AFM measurements of fixed tissues in an aqueous environment, but in a small area of interest, were confirmed in the definitely bigger area of interest using SEM imaging in non-physiological conditions after gold sputtering. Previous literature indicated the formation of nanoscale protuberances on the surface of cultured endothelial cells using AFM and defined them as clusters for trapping leukocytes during inflammation, rich in inter alia ICAM-1 [3]. Indeed, the ICAM-1 overexpression of ECs upon TNF-induced inflammation has also been demonstrated in this study (Fig. 6C), as well as diminished expression of the ICAM-1 in response to Rac1 inhibition by NSC23766 (Fig. 6D). It is supposed, that the regulatory mechanism of TNF/Rac1 on ICAM-1 expression in endothelium could be similar to the action of TNF in the lung airway epithelial cells via a Rac1–ROS–NF-ĸB–ICAM-1-linked cascade [22], especially that regulatory role of Rac1 to NF-ĸB, and in consequences the expression of ICAM-1 in the cultured endothelial cell were reported [23].

TNF-induced inflammation of endothelial cells is associated with the formation of lipid droplets, augmented cortical stiffness, as well as nanostructural endothelial plasma membrane remodeling. All these phenomena seem to be Rac-1 dependent, but further studies are need to understand in-depth mechanisms linking and explaining these phenomena, on subcellular level, or in vivo [48].

The important limitation of the present work explaining our experimental approach was that in TNF-stimulated aorta en face, newly formed LDs in the endothelial layer were rapidly metabolized in contrast to endothelial cells in culture maintaining LDs for a longer time [8, 9]. Thus, to characterize LDs in the isolated vessel setup, it was necessary to use atglistatin to block the lipolysis of LDs. Importantly, the observed effect of the NSC23766 inhibitor on F-actin polymerization (Fig. 4) and ICAM-1 protein expression (Fig. 6) was independent of the use of atglistatin suggesting that atglistatin was selectively responsible for blocking lipolysis of LDs, but did not interfere with the mutual correlations of LDs formation, the activity of Rac1 protein, nanomechanical and nanostructural alterations linked to vascular inflammation induced by TNF. Of note, despite these limitations, we claim that further studies in the isolated vessels or in vivo are instrumental to provide a novel insight into the pathophysiological role LD and fatty acids liberated from the LDs formed in ECs, either to underlying tissue, or back into the circulation.

In conclusion, we underscored the central and indispensable role of Rac1 in TNF-induced inflammation of ECs in the isolated murine aorta. Our results indicated that LDs formation was dynamically regulated by both Rac1 and ATGL. Activation of Rac1 was shown to represent a central pathway orchestrating LDs formation with nanomechanical and nanostructural alterations of the TNF-induced inflammation of ECs inside isolated blood vessels and thus may provide a therapeutic target for reducing vascular inflammation.

## Supplementary Information

Below is the link to the electronic supplementary material.

Supplementary file1 (DOCX 3187 KB)

## Data Availability

Not applicable.
