# Supplementary material for: Rac1 regulates lipid droplets formation, nanomechanical, and nanostructural changes induced by TNF in vascular endothelium in the isolated murine aorta
Source: Cell Mol Life Sci. 2022 May 27;79(6):317. doi: 10.1007/s00018-022-04362-7 (PMC9142475; doi:10.1007/s00018-022-04362-7)
Supplement: Supplementary file 1 — Supplementary file1 (DOCX 3187 KB) [file 18_2022_4362_MOESM1_ESM.docx]

***Supplementary Information***


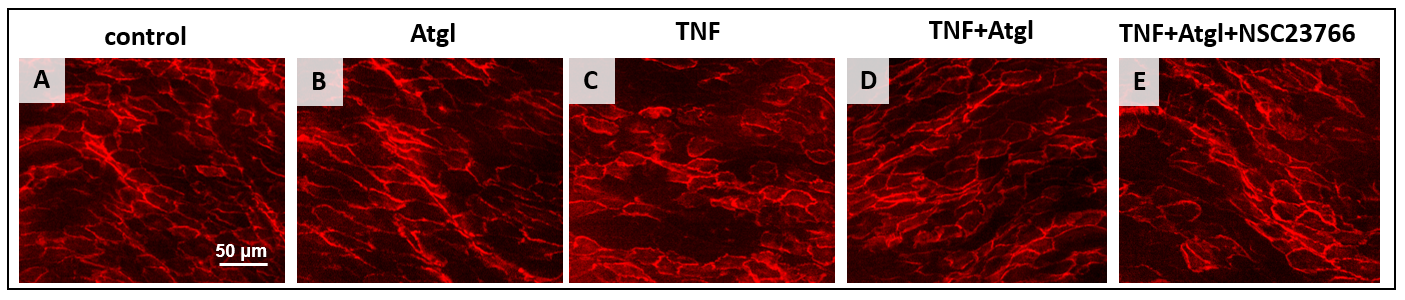


***Fig. S1.*** *Representative microphotographs of immunostaining of control en face aorta (A), aorta treated with Atgl (B; 10 µM, 48h), TNF (C; 10 ng/ml, 48h), TNF+Atgl (D; 10 ng/ml and 10 µM, respectively, 48h), and aorta treated with TNF+Atgl+NSC23766 (E; 10 ng/ml, 10 µM and 50 µM, respectively, 48h). Red fluorescence originate from PECAM-1. The visual cobble-stone pattern of endothelium indicated intact endothelial layer in isolated blood vessel in all studied groups.*


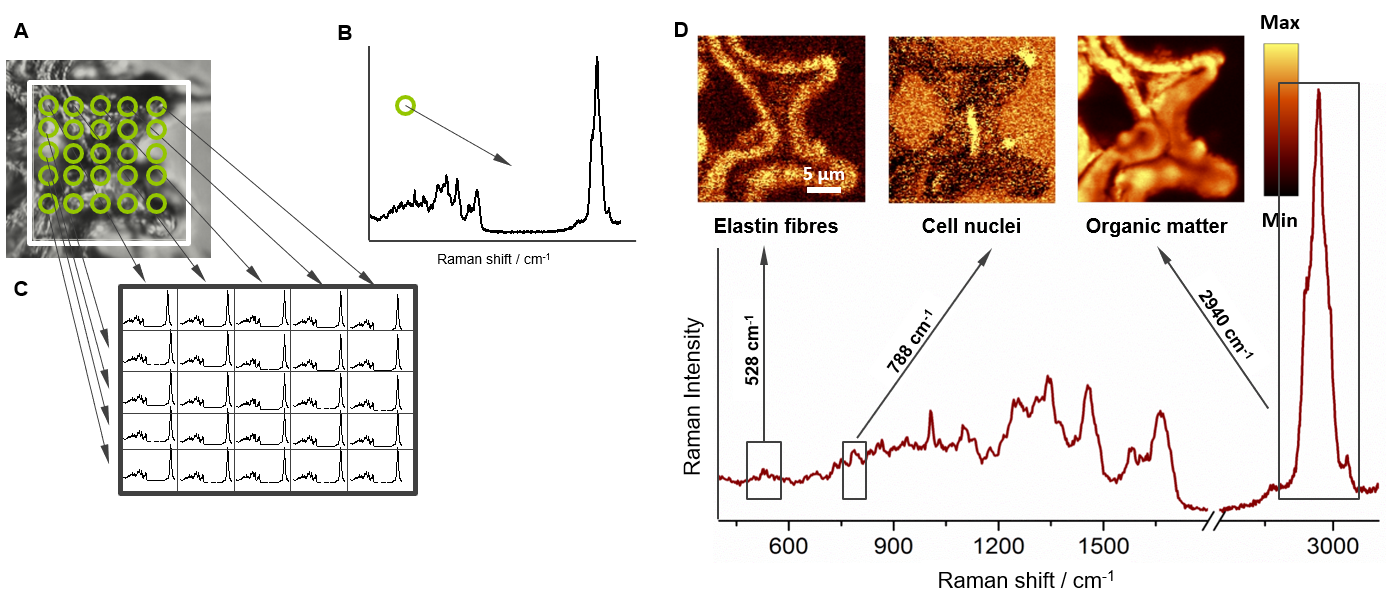


***Fig. S2.*** *The principles of Raman imaging. Raman images are recorded by scanning the sample in x and y direction in such a way that a complete Raman spectrum is obtained at every voxel. Therefore, Raman imaging results in set of Raman spectra containing chemical information about the studied sample. Finally, the intensity of the “marker” (characteristic for a given component) band is calculated in every Raman spectrum and, after color-coding, it becomes a visual representation of distribution of the studied component in the sample (please see below, the scheme visualizing the basics of Raman imaging). Thus, the images of the distribution of cellular structures including cell nuclei or lipid droplets may not be homogeneous, as the intensity of the Raman signal and the marker band may differ from each other in different places. Raman spectroscopy does not only provide information about the presence / location / distribution of cellular components in the sample, but can be used to identify chemical fingerprints of distinct cellular components, and track theirs alterations in biochemical composition.*


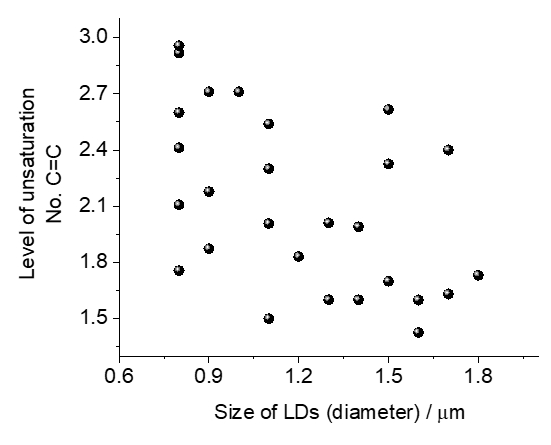


***Fig. S3.*** *A slight trend pointing to the relationship between endothelial LDs individual level of unsaturation with their sizes. LDs having a smaller diameter then 800 nm were not included in the analysis as their size is less than the diffraction limit.*


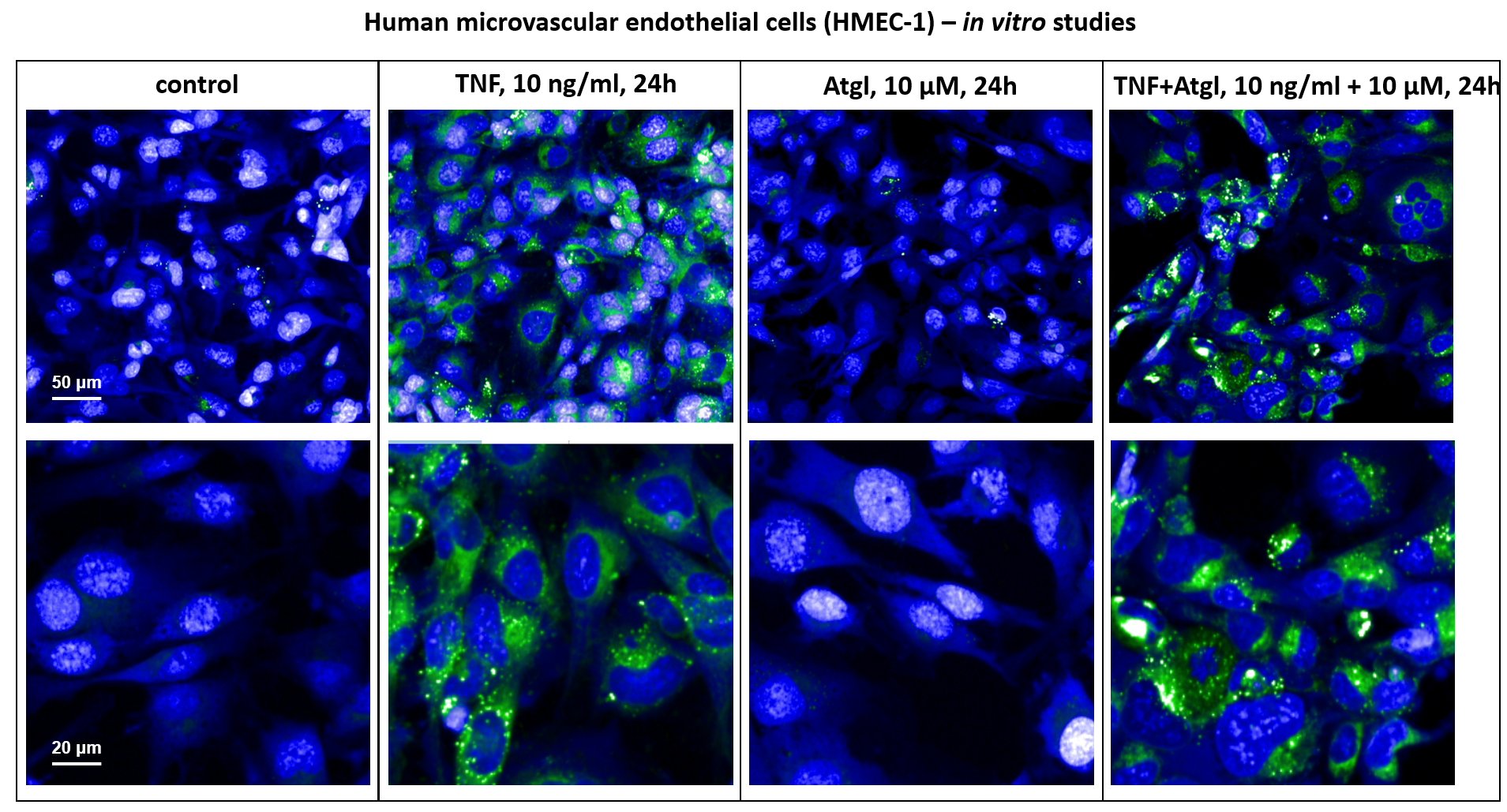


***Fig. S4.*** *Formation of LDs in TNF-stimulated HMEC-1 in vitro in the absence and presence of atglistatin. Representative fluorescence images of control, atglistatin (10 µM), TNF (10 ng/ml), or TNF+Atgl-treated HMEC-1 (10 ng/ml + 10 µM, respectively, 24h) showing the distribution of nuclei (blue, Hoechst 33342), and lipid droplets (green, BODYPY 493/503).*

**Detailed materials and methods**

**Sample preparation**

All experimental procedures involving animals were conducted according to the Guidelines for Animal Care and Treatment of the European Communities and the Guide for the Care and Use of Laboratory Animals published by the US National Institutes of Health (NIH Publication No. 85-23, revised 1996). All procedures were approved by the Local Ethical Committee on Animal Experiments.

C57/BL/6J mice (wild type) at the age of 10-15 weeks were anesthetized by intraperitoneal injection (*i.p.*) of a mixture consisting of ketamine and xylazine in the dose of 100 mg ketamine/10 mg xylazine/kg body weight (all studies excluding AFM measurements). The chest was opened, and the thoracic aorta was quickly removed and transferred into Krebs–Henseleit buffer. Subsequently, aorta were cleaned from surrounding tissue, cut into rings and transferred into medium (minimal essential medium (MEM) with the addition of 1% MEM vitamins, 1% antibiotics (penicillin 10,000 U/mL and streptomycin 10,000 μg/mL), 1% non-essential amino acids, and 20% fetal bovine serum), in the absence or presence of murine TNF (10 ng/ml, 24 and 48h; Sigma Aldrich), atglistatin (10 µM, 48h; Cayman Chemical Company; ; denoted in the manuscript as Atgl), or NSC23766 trichydrochloride (50 µM, 24 and 48h; Sigma Aldrich; denoted in the manuscript as NSC23766). Aorta preparations were incubated at 37°C and 5% CO_2_.

**Raman imaging of aorta *en face***

For Raman measurements aorta *en face* treated with TNF in the presence of atglistatin (10 ng/ml+10µM, 48h) was used (N=5). The resected and split-open arteries were tightly glued to the Cell-Tak®-coated calcium fluoride surface. Subsequently, the tissue was preserved by a 10-min soak in 4% buffered formalin.

Raman imaging was carried out using a WITec Confocal Raman Imaging system (WITec alpha300, Ulm, Germany) supplied with a UHTS 300 spectrograph (600 grooves·mm^−1^ grating, resolution of 3 cm^−1^) and a CCD detector (Andor, DU401A-BV-352). The air-cooled solid state laser with the excitation wavelength of 532 nm was coupled to the microscope *via* an optical fiber with a diameter of 50 μm. Raman spectra of tissues were collected with the application of a 63× water immersive objective (Zeiss Fluor, NA = 1.0), using maximum laser power at the sample position (*ca.* 30 mW) and 0.4s exposure time per spectrum. The nominal minimal lateral and depth resolution for our setup is 0.32 and 0.53 μm, respectively, and sampling density of 0.40-0.50 and 0.5-1.0 μm in x/y and z direction, respectively, was used. Depth profiling of the tissue was obtained by multiple imaging of the same line in several layers of the sample. The distribution images collected at different depths present the relative intensity of a studied component in the tissue. Data matrices were analysed using a WITec Project software (background subtraction using a polynomial of degree 2 and the automatic removal of cosmic rays). For study of heterogeneity of LDs observed in *in situ* endothelial cells within isolated blood vessels, the single Raman spectra were extracted from the centre of each LD, and then averaged. The averaged spectra presented in figure 3, were normalized in the OPUS program using vector normalization option. After calculating the average y value of spectra in the range of 500-3100 cm^-1^, the average value calculated will then be subtracted from the spectrum. This is followed by calculating the sum of squares of all y values, and the respective spectrum is divided by the square root of this sum. The vector norm of the resulting spectrum is 1. The OPUS 7.2 program was used for calculations of the integral intensity of the bands at *ca.* 1660 and 1445 cm^-1^ in the 1563-1712 and 1394-1505 cm^-1^ spectral ranges, respectively. Integration was performed using method D, OPUS 7.2: the integral was defined by the wavenumber limits and the horizontal baseline determined by a chosen baseline point.

**Immunostaining of aorta *en face*: LDs and PECAM-1 detection**

Fixed samples (by a 15-min soak in 4% paraformaldehyde) were blocked with TNB blocking buffer (0.1 M Tris-HCl pH 7.5, 0.15 M NaCl, and 0.5% (w/v) blocking reagent; PerkinElmer FP1020) for 3-4h, and then incubated with anti-PECAM-1 antibody (EMD Millipore, MAB1398Z, 1:250) diluted in TNB blocking buffer overnight at 4°C. As secondary antibody, Alexa Fluor 647 nm goat-anti-rabbit (Jackson Immuno Research; 3:600) was used at room temperature for 3 hours. BODIPY 493/503 diluted in PBS at the final concentration of 0.1 mg/ml was applied for 1h to delineate LD, and Hoechst 33258 (Sigma; 1:1000) was used to highlight nuclei. Samples were measured by a 40× magnification objective on CQ1 Confocal Quantitative Image Cytometer (Yokogawa) or Nikon inverted microscope Ti-E using a PlanApo 100×/1.4 Oil DIC objective. Overall, for fluorescence detection of LDs/PECAM-1 the N=10, N=5, N=10, N=5 and N=5 for control aorta and aorta treated with Atgl (10 µM, 48h), TNF (10 ng/ml, 48h), TNF+Atgl (10 ng/ml+10 µM, 48h), TNF+Atgli+NSC23766 (10 ng/ml+10 µM+50 µM, 48h, respectively, were studied. N denotes number of mice. As a negative control for immunolabeling, primary antibody against PECAM-1 was omitted.

**F-actin fluorescence visualisation**

Fixed samples (by a 10-min soak in 4% paraformaldehyde) were permeabilized with TritonX 100 (1min) and gently washed with PBS afterwards. Samples of aorta were preincubated with normal goat serum (30 min), and then incubated with Phalloidin–Tetramethylrhodamine B isothiocyanate (Phalloidine-TRITC, Sigma Aldrich) in a dilution of 1:100 for 60 min. Then Hoechst 33258 (Sigma; 1:1000; 15 min) was used to highlight nuclei. Fluorescence images of cortical F-actin were acquired with an A1-Si Nikon (Japan) confocal laser scanning system built onto a Nikon inverted microscope Ti-E using a PlanApo 100×/1.4 Oil DIC objective. The images were acquired at a resolution of 102461024. Cell nuclei and F-actin were excited with 405 and 561 nm diode lasers, respectively. Colour-codded pictures were reconstructed using NIS-Elements AR 3.2 software.

Overall, for fluorescence visualisation of F-actin the N=3 for all groups: control, NSC23766 (50 µM, 24h), TNF (10 ng/ml, 24h), TNF+NSC23766 (10 ng/ml and 50 µM, respectively, 24h), Atgl (10 µM, 24h), TNF+Atgl (10 ng/ml and 10 µM, respectively, 24h), TNF+Atgl+NSC23766 (10 ng/ml, 10 µM and 50 µM, respectively, 24h) were studied. The F-actin-based fluorescence was quantified by counting the mean intensity of Phalloidin signal using ImageJ software (National Institutes of Health, USA).

**Stiffness measurements**

For stiffness measurements the endothelium inside non-fixed split-open aorta, tightly glued to the Cell-Tak®-coated microscopic glasses was used. The cortical stiffness of the endothelium was determined using an Atomic Force Microscope (AFM; MultiMode SPM, Bruker, Germany) equipped with a feedback-controlled heating device (Nanoscope Heater Controller; Digital Instruments, Veeco, USA). To determine exclusively the stiffness of the endothelial cell cortex, soft triangular cantilevers (Novascan, USA) with a nominal spring constant of 0.03 N/m and a polystyrene sphere (10 µm) as a tip were used, whereas a ramp size of 2 µm and a trigger threshold of 100 nm were chosen. Overall, for stiffness measurements the N=5 mice were studied within each studied group: control, NSC23766 (50 µM), TNF (10 ng/ml), or TNF+NSC23766 (10 ng/nl+50 µM, respectively), after 24h of incubation. For time-dependent stiffness measurements the N=8, N=5, N=6, N=6, N=4 for control mice and mice treated with TNF in the concentration of 10 ng/ml for 1-2, 5-6, 24, or 48h, respectively, were studied. The analysis was performed using the Protein Unfolding and Nano-Indentation Analysis Software (PUNIAS), in the mode for “nanoindentation”.

**AFM and SEM imaging of aorta *en face***

AFM imaging was carried out using a WITec Confocal Raman Imaging system (WITec alpha300, Ulm, Germany). AFM imaging of topography and phase were performed in the tapping (AC) mode with the force modulation probes (k=0.2 N/m, WITec) using the 20× objective (NA = 0.5, Olympus). For AFM imaging of topography and phase, the samples were fixed by a 10-min soak in 4% buffered formalin, then dried and measured in air. Subsequently after AFM imaging, the same samples of aorta *en face* were measured by SEM.

SEM measurements of dough were performed on a Tescan Vega3 LM microscope with LaB 6 cathode. Samples were coated with a thin gold layer in Quorum Q150T sputter coater. The accelerating voltage was set to 30 kV.

Overall, for AFM and SEM imaging the N=3 for each group: control, NSC23766 (50 µM), TNF (10 ng/ml), or TNF+NSC23766 (10 ng/nl+50 µM, respectively), after 24h of incubation were studied.

**Immunostaining** **of ICAM-1expression in cross-section of aorta**

Rings of the aorta were embedded in the OCT medium (Thermo) and frozen at −80°C using Leica CM1920 automatic cryostat (Leica, Wetzlar, Germany). The 5 μm thick cross-section slides were put on the microscopic glasses coated with poly-L-lysine. Then, slices of aorta were fixed with for 10 min in 4% buffered formalin (Merck) and used for immunostaining of ICAM-1.

Before staining aortic rings were permeabilized with TritonX 100. Aortic rings were preincubated with 5% normal goat serum (Jackson Immuno Research) and 2% dry milk in PBS, then immunostained using rat-anti-mouse ICAM-1 (eBioscience; 1:200) primary antibody overnight. As secondary antibodies Cy3-conjugated goat-anti-rat (Jackson Immuno Research; 1:300) was usedfor 30 min. Cell nuclei were visualized by Hoechst 33258 (Sigma; 1:1000) solution and unspecific fluorescence of aortic elastic fibres were used as a background counterstaining channel. Images were acquired using an AxioCam HRm digital monochromatic camera and an AxioObserver.D1 inverted fluorescent microscope (Carl Zeiss). The ICAM-1 fluorescence was quantified by counting the area of the intensity of ICAM-1 signal vs. area of whole tissue using ImageJ software (National Institutes of Health, USA).

Overall, for immunostaining of ICAM-1expression the N=6, N=4, N=6, N=4, N=3, N=3, and N=3 for control, NSC23766 (50 µM), TNF (10 ng/ml), TNF+NSC23766 (10 ng/nl+50 µM, respectively) atglistatin (10 µM), TNF+Atgl (10 ng/ml and 10 µM, respectively), and TNF+Atgl+NSC23766 (10 ng/ml, 10 µM and 50 µM, respectively), after 24h of incubation, were studied.

**Cell culture**

HMEC-1 cells (human dermal microvascular endothelial cells; American Type Culture Collection company, USA) were cultured in complete MCDB131 medium (Gibco Life Technologies) supplemented with 10 mM L-glutamine (Gibco Life Technologies), 1 μg/ml hydrocortisone (Sigma Aldrich), 10 mg/ml epidermal growth factor (EGF, Sigma Aldrich), 10% fetal bovine serum (FBS, Gibco Life Technologies) and antibiotic antimycotic solution (AAS with 10.000 U penicillin, 10 mg streptomycin and 25 μg amphotericin B per ml) and maintained at 37°C in atmosphere of air with 5% CO_2_ in a cell culture incubator. After 24 hours of incubation cells were rinsed twice with phosphate buffered saline (PBS, pH 7.4, Gibco Life Technologies) and exposed to TNF (10 ng/ml), atglistatin (10 µM), or TNF together with atglistatin for 24 hours. HMEC-1 cells fixed with 2.5% solution of glutaraldehyde in PBS for 4 minutes. BODIPY 493/503 diluted in PBS at the final concentration of 0.1 mg/ml was applied for 1h to delineate LD, and Hoechst 33258 (Sigma; 1:1000) was used to highlight nuclei. HMEC-1 cells were measured by a AxioCam HRm digital monochromatic camera and an AxioObserver.D1 inverted fluorescent microscope (Carl Zeiss).

**Statistical analysis**

All data were considered significant if *p* ≤ 0.05. After testing for normal distribution, the two-sample t-test or ANOVA (Raman), or the two-sample t-test or Mann–Whitney test ^48^ was performed. All values are given as mean ± SEM.
